# Aortic velocity measurements derived from phase‐contrast MRI are influenced by a cardiac implantable electronic device in both adult and pediatric human subjects

**DOI:** 10.1002/mrm.30399

**Published:** 2024-12-06

**Authors:** Huili Yang, Oluyemi B. Aboyewa, Gregory Webster, Dhaivat Shah, Laleh Golestanirad, Justin J. Baraboo, Michael Markl, Jeremy D. Collins, Bradley P. Knight, KyungPyo Hong, Amit R. Patel, Daniel C. Lee, Daniel Kim

**Affiliations:** ^1^ Department of Radiology Northwestern University Feinberg School of Medicine Chicago Illinois USA; ^2^ Department of Biomedical Engineering, McCormick School of Engineering Northwestern University Evanston Illinois USA; ^3^ Division of Cardiology, Department of Pediatrics Ann & Robert H. Lurie Children's Hospital Chicago Illinois USA; ^4^ Department of Radiology Mayo Clinic Rochester 55905 Minnesota USA; ^5^ Department of Medicine (Cardiology) Northwestern University Feinberg School of Medicine Chicago Illinois USA; ^6^ Department of Medicine (Cardiovascular Division) University of Virginia School of Medicine Charlottesville Virginia USA

**Keywords:** aortic flow, CIED, phase‐contrast MRI, real‐time MRI

## Abstract

**Purpose:**

Overall there is a lack of evidence on the accuracy and precision of phase‐contrast (PC) MRI in patients with cardiac implantable electronic devices (CIEDs). The purpose of this study is to determine whether aortic velocity measurements are influenced by a CIED.

**Methods:**

We scanned 21 adult patients and 8 pediatric volunteers using clinical standard PC and real‐time PC (rt‐PC) sequences with and without a CIED generator taped on human subjects (below the left clavicle for adults and children; also on the abdomen for children) to mimic image artifacts. Peak and mean velocities above the aortic valve were calculated.

**Results:**

The Bland–Altman analyses on peak velocity measurements in pediatric subjects showed that both the accuracy and precision worsen as the distance between the CIED and aortic valve decreases (i.e., from abdomen to below the left clavicle). Specifically, both the bias and the coefficient of variation (CV) for both clinical PC and rt‐PC increased from the abdominal position (clinical: bias = −1.1%, CV = 4.3%; rt‐PC: bias = −0.3%, CV = 3.4%) to the clavicle position (clinical: bias = −4.0%, CV = 8.1%; rt‐PC: bias = 8.2%, CV = 7.3%). A similar trend was observed for mean velocity. The mean difference in peak and mean velocity measurements between rt‐PC with CIED (either position) and clinical standard PC with no CIED was within 7.5%. In adult patients, the mean difference between rt‐PC with CIED and clinical standard PC with CIED in peak velocity was 6.9%, and the CV was 7.9%.

**Conclusion:**

This study demonstrates that aortic velocity measurements are influenced by CIED in both adult and pediatric subjects.

## 1 INTRODUCTION

Two‐dimensional (2D) phase contrast (PC) MRI is used routinely in clinical practice for quantitative assessment of blood flow in patients with a variety of cardiovascular diseases.^1^, ^2^, ^3^ Patients with cardiac implantable electronic devices (CIEDs) with valvular heart disease might benefit from PC MRI. Although a growing list of studies has addressed MR safety in CIED patients,^4^, ^5^, ^6^, ^7^, ^8^, ^9^ to our knowledge, only two published studies have tested PC MRI in patients with CIEDs.^6^, ^10^ Most off‐resonance artifacts (e.g., signal voids) originate from the implantable pulse generator (CIED), with lesser contribution from the leads of the device.^11^ Of note, the signal loss (i.e., intravoxel dephasing) caused by the generator reduces PC precision, while the field distortion (i.e., inaccurate phase correction) reduces PC accuracy. Regrettably, there is overall a lack of studies describing the influence of CIEDs on the accuracy and precision of hemodynamic parameters derived from 2D PC MRI.

The presence of a CIED generator is the predictable source of image artifacts in 2D PC MRI. In adults with CIEDs, the CIED generator is typically implanted below the left clavicle, approximately 10–15 cm away from the heart. In children, the CIED is typically implanted in the abdomen, with leads sewn onto the epicardium. Teenagers with CIEDs may have an endocardial lead system, where the CIED is implanted below the left clavicle. In general, the CIED‐induced, off‐resonance effects on the heart are greater in children due to the smaller distance between the CIED and the heart. Hence, PC MRI would be more sensitive to CIED presence in pediatric CIED patients than in adults.

Other sources of unpredictable image artifacts in CIED patients include arrhythmias and dyspnea, both of which are common in this patient population. One approach to reducing these artifacts is performing real‐time 2D PC (rt‐PC) MRI.^12^, ^13^, ^14^, ^15^, ^16^, ^17^, ^18^, ^19^, ^20^, ^21^, ^22^, ^23^ Real‐time imaging permits free‐breathing scanning, eliminating the need for breath‐holding, which is required for clinical standard 2D PC MRI. Additionally, real‐time imaging makes PC MRI less sensitive to arrhythmia by eliminating segmented k‐space sampling over multiple heartbeats, the mode of scanning for clinical 2D PC MRI. Rt‐PC provides additional benefits over clinical PC MRI, such as beat‐to‐beat evaluation of hemodynamics^23^ and shorter scan times.^12^ To our knowledge, no study has investigated the relative accuracy and precision of PC MRI in both pediatric and adult subjects with CIEDs. Yang et al. recently developed a free‐breathing, rt‐PC sequence with an effective temporal resolution of 25 ms,^23^ by amplifying GRASP (golden‐angle radial sparse parallel)^24^ with view sharing,^25^, ^26^ and k‐space‐weighted image contrast filtering,^27^ but this sequence has not been evaluated in patients with CIEDs. The aim of this study is to determine whether aortic velocity measurements derived from 2D clinical standard PC and rt‐PC pulse sequences are influenced by a CIED in pediatric and adult subjects.

## 2 METHODS

In this study, accuracy is defined as the bias relative to clinical standard 2D PC MRI in the Bland–Altman analysis; precision is defined as the coefficient of variation (CV), calculated as a percentage of the standard deviation of differences relative to the mean of measurements in the Bland–Altman analysis.

### 2.1 Taping an CIED to mimic image artifacts

For our study, we evaluated the accuracy and precision of rt‐PC in human subjects with an implantable cardioverter‐defibrillator (ICD) generator (Vitality AVT Model A155, GUIDANT; Boston Scientific, Natick, MA, USA) taped to the chest or abdomen to mimic image artifacts as if in situ (see Figures S2 and S3 for anatomic locations where ICD was taped). This is an established approach to test pulse sequences^28^, ^29^ with several advantages.

### 2.2 Phantom experiment

A pulsatile flow phantom (a U‐shaped polyvinyl chloride pipe with an inner size of 21 mm representing a simplified aorta; see Figure S1) was scanned with and without ICD positioned about 12 cm away from the flow tube ipsilateral using clinical PC and rt‐PC, to evaluate the influence of CIED on velocity and flow measurements. A pneumatically driven ventricular assist device controlled by a pressure pump control unit (MEDOS, Germany)^30^ was attached to the flow phantom and generated pulsatile flow through the phantom at a frequency of 60 beats per minute. Water doped with gadolinium‐based contrast agent was used as the fluid. Electrocardiogram gating was performed using a synchronized trigger signal generated by the pump control unit. Pulse sequence parameters for the phantom experiment were similar to those used in in vivo experiments (see Section 2.5), except a lower velocity encoding (60 cm/s) was used, because this particular ventricular assist device pump was unable to generate higher velocity without causing ghosting artifacts in electrocardiogram‐gated segmented PC MRI. The flow phantom experiment was repeated with flow pump on and off while maintaining identical imaging parameters and prescan settings, to correct for background phase offset.^31^

### 2.3 Study population

This study was conducted in accordance with protocols approved by our institutional review board and was compliant with the Health Insurance Portability and Accountability Act. All subjects and/or guardians consented in writing to participate in this study.

In the first experiment testing for accuracy and precision, we recruited 8 pediatric healthy volunteers (2 males and 6 females, mean age = 13.5 ± 1.4 years; see Table S1) with no prior history of heart disease and performed clinical PC and rt‐PC without gadolinium or general anesthesia. We performed both PC pulse sequences at the aortic valve plane with no ICD, with the ICD taped below the left clavicle (simulating a transvenous CIED), and with the ICD taped on the anterior abdomen (simulating an epicardial CIED).

In the second experiment testing for relative accuracy, we added PC MRIs slightly above the aortic valve level to an ongoing parent study for 21 adult patients (12 males and 9 females, mean age = 49.9 ± 14.4 years; see Table S1 for detailed clinical profiles). Study participants without coronary artery disease before MRI evaluation were included based on referral for a clinical stress cardiovascular MRI. The ICD generator was taped below the left clavicle (˜12 cm from the heart). In all patients, clinical PC was performed during breath‐holding, whereas rt‐PC was performed during free breathing. Both PC sequences were performed approximately 5–10 min after administration of 0.20 mmol/kg of gadobutrol (Gadavist; Bayer HealthCare Pharmaceuticals, Whippany, NJ, USA). Because our rt‐PC data were reconstructed offline, the clinical PC was performed first to visually identify phase wrapping inline. In the adult experiment, we were unable to perform clinical PC without ICD as reference. This was because the parent study, which lasted 60–75 min and involved administration of adenosine and gadolinium, required the ICD to remain on the subjects throughout the MRI experiment.

### 2.4 MRI hardware

MRI was performed on one 1.5T whole‐body MRI scanner (MAGNETOM Aera; Siemens Healthineers, Erlangen, Germany) equipped with a gradient system capable of achieving a maximum gradient strength of 45 mT m^−1^ and a maximum slew rate of 200 T m^−1^ s^−1^. A body coil was used for RF excitation, and standard body flex, and spine coil arrays (20–26 elements) were used for signal reception.

### 2.5 Pulse sequence

For relevant imaging parameters for clinical PC and rt‐PC, please see Table S2. Note that PC MRI is based on gradient‐echo readout. As such, using a low flip angle (≤ 20°) achieves a specific absorption rate significantly less than 2.0 kW/kg, the Food and Drug Administration limit for scanning patients with CIEDs.^32^

### 2.6 Image analysis

Semi‐automated image processing (phase unwrapping, background phase correction, and region of interest [ROI] contouring) was performed using cvi42 v5.14 (Circle Cardiovascular Imaging, Calgary, Canada) to calculate hemodynamic parameters derived from clinical PC and rt‐PC data. For correcting the background phase offset in the phantom data, we subtracted the phase difference of flow pump off data from the phase of flow pump on data. For correcting the background phase offset in vivo, we used first‐order fitting for clinical PC sampled with Cartesian k‐space sampling, whereas second‐order fitting for rt‐PC was used to account for the nonlinear phase offsets induced by non‐Cartesian k‐space sampling. For rt‐PC analysis, we measured the peak and mean velocities at peak systole for all cardiac cycles (3–7 cardiac cycles per subject) and reported the median value. We chose not to analyze forward and backward volumes due to the high number of cardiac frames (= 10 353). In select cases corresponding to the Figures 2 and 4, we additionally measured flow through one full cardiac cycle after performing linear interpolation, to match temporal resolution and aligning the curves using cross‐correlation.

### 2.7 Statistical analysis

We tested for variable normality using the Shapiro–Wilk test. Bland–Altman analysis was performed on peak and mean velocities to assess the level of agreement between measurements. The CV was calculated. For the adult study, paired two‐tailed t‐tests (Wilcoxon signed‐rank tests if not normally distributed) were used to detect any significant differences between results from clinical PC and rt‐PC. For the pediatric study, one‐way analysis of variation (Kruskal‐Wallis if not normally distributed) with Bonferroni correction was conducted to detect any significant differences among results from clinical PC without ICD (reference), clinical PC with ICD, rt‐PC without ICD, and rt‐PC with ICD. A *p*‐value less than 0.05 was considered statistically significant for all tests performed.

## 3 RESULTS

**FIGURE 1:**
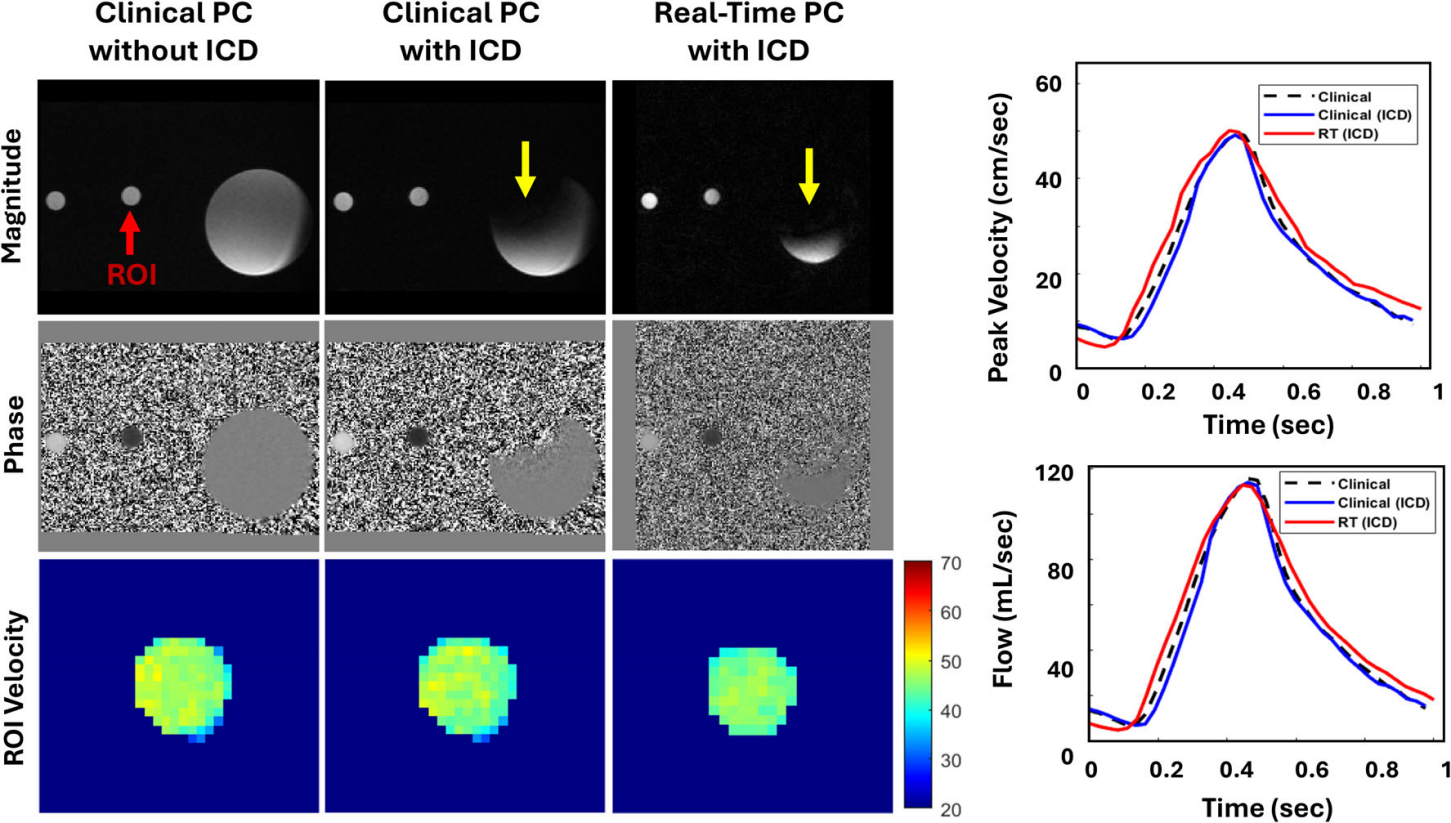
The flow phantom experiment for evaluating the influence of an implantable cardioverter‐defibrillator (ICD) on velocity and flow measurements. The flow tube was approximately 12 cm away from the ICD. Phase‐difference images are displayed with grayscale ranging from −π to π. For real‐time phase contrast (PC), three beats were averaged to generate the two‐dimensional velocity profile within the region of interest (ROI) at peak systole and the peak velocity and flow‐time curves. Yellow arrows point to signal voids caused by the ICD. For the corresponding video display, see Video S1. RT, real time.

Figure 1 shows the magnitude and PC images, 2D velocity profile within the ROI at peak systole, and the corresponding peak velocity and flow curves from the flow phantom scans with and without ICD positioned 12 cm away from the ROI. The ICD did not cause significant signal loss in the ROI. A difference in the 2D velocity profile was observed between with and without ICD in clinical PC. Clinical PC with ICD produced flow and velocity at peak systole in the ROI within −1.47% and −1.09%, respectively, compared with clinical PC without ICD (reference). Similarly, Rt‐PC with ICD produced flow and velocity at peak systole in the ROI within −2.43% and 0.81%, respectively, compared with the reference. The normalized root mean squared error (NRMSE) in peak velocity for all time points was 3.95% and 7.05% for clinical PC with ICD and rt‐PC with ICD, respectively; the corresponding NRMSE in flow for all time points was 3.69% and 5.59%. For dynamic display of images in Figure 1, see Video S1.

**FIGURE 2:**
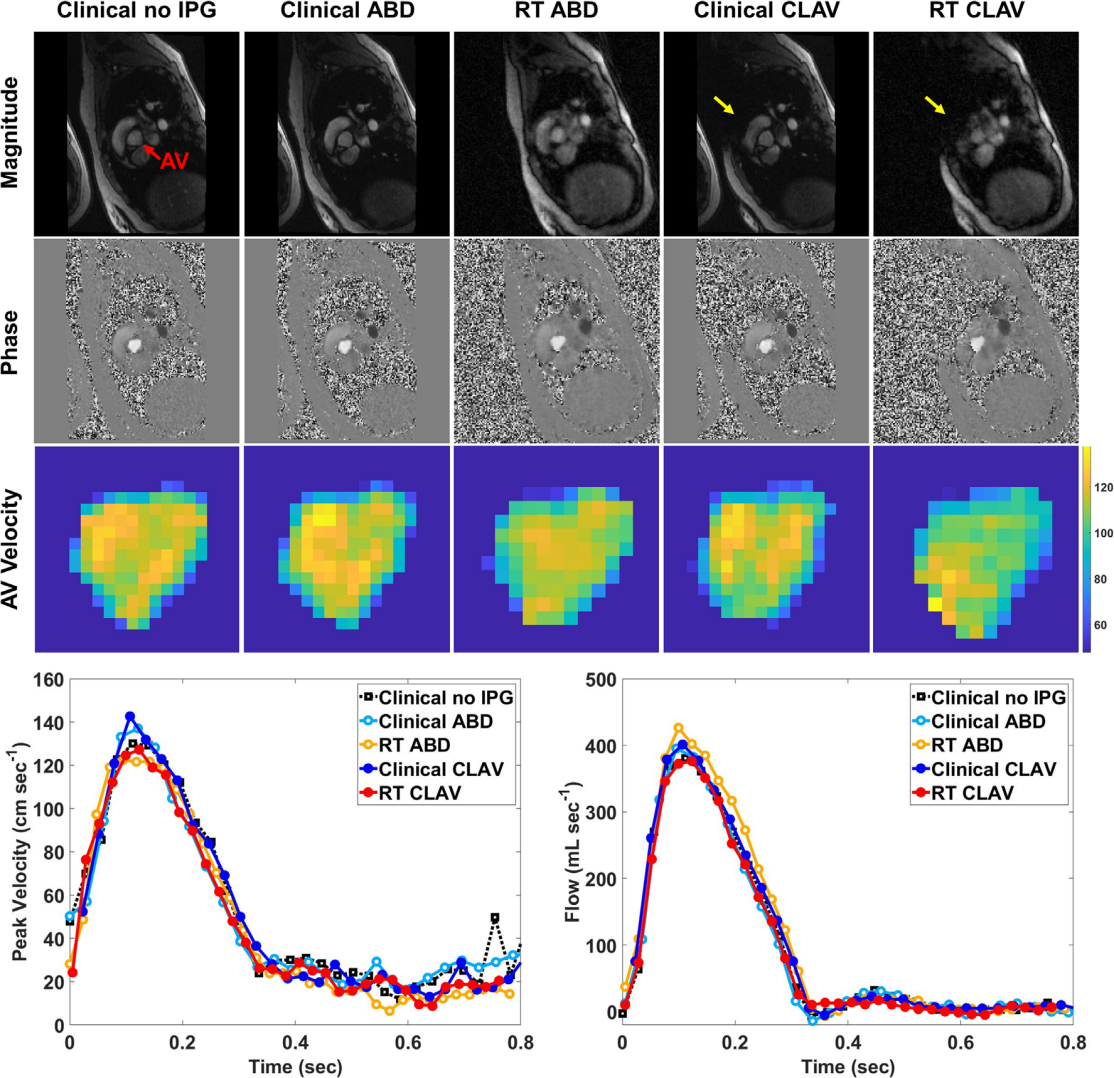
The magnitude images, phase‐difference images, and two‐dimensional aortic velocity profiles at peak systole of a representative pediatric subject without (*Column 1*) and with implantable cardioverter‐defibrillator (ICD) taped on the abdomen (*Columns 2–3*) and below the left clavicle (*Columns 4–5*). The corresponding peak velocity and flow‐time curves above the aortic valve are shown. Phase images are displayed with grayscale ranging from –π to π. Yellow arrows point to signal voids caused by the ICD. For the corresponding video display, see Video S2. ABD, with ICD taped on the abdomen; AV, aortic valve; CLAV, with ICD taped below the left clavicle; RT, real time.

Figure 2 shows the magnitude and PC images, 2D aortic velocity profile at peak systole, and the corresponding peak velocity and flow measurements of a representative pediatric volunteer with and without ICD taped on the abdomen and below the left clavicle. A difference in the 2D velocity profile was observed among without ICD, with ICD taped on the abdomen, and with ICD taped below the left clavicle in clinical PC. The difference in the 2D velocity profile between with ICD taped on the abdomen and below the left clavicle was more prominent in rt‐PC than clinical PC. For ICD taped on the abdomen, the NRMSE in peak velocity for all time points was 5.79% and 8.27% for clinical PC with ICD and rt‐PC with ICD, respectively; the corresponding NRMSE in flow for all time points was 2.45% and 5.63%. For ICD taped below the left clavicle, the NRMSE in peak velocity for all time points was 6.52% and 7.31% for clinical PC with ICD and rt‐PC with ICD, respectively; the corresponding NRMSE in flow for all time points was 3.12% and 2.31%. For dynamic display of images in Figure 2, see Video S2.

**FIGURE 3:**
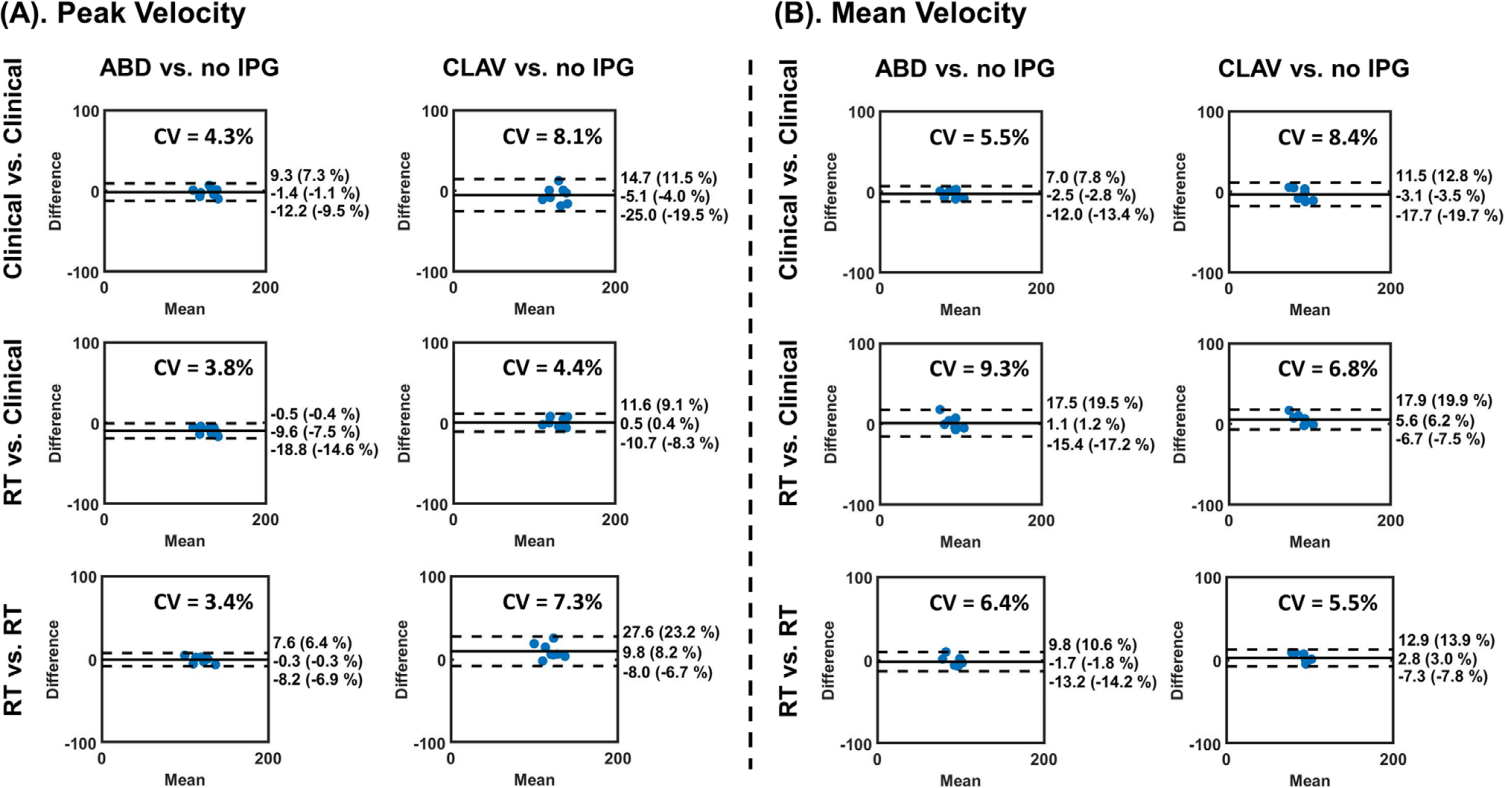
Scatter plots representing the Bland–Altman analysis on peak velocity (A) and mean velocity (B) measurements at peak systole with implantable cardioverter‐defibrillator (ICD) taped on the abdomen (*left column*) or below the left clavicle (*right column*) in children. *Row 1*: Clinical standard versus clinical standard. Row 2: Real‐time phase contrast (rt‐PC) versus clinical standard. *Row 3*: Rt‐PC versus rt‐PC. Units: cm/s. ABD, with ICD taped on the abdomen; CLAV, with ICD taped below the left clavicle; CV, coefficient of variation; IPG, implantable pulse generator; RT, real time.

Figure 3 shows scatter plots illustrating the Bland–Altman analysis on peak and mean velocity measurements at peak systole, including all pediatric subjects with and without ICD. For clinical PC, taping an ICD on the abdomen revealed a small bias (−1.1%) and a small CV (4.3%) in peak velocity, and a small bias (−2.8%) and a small CV (5.5%) in mean velocity. With this as a benchmark, taping an ICD below the left clavicle increases both the bias (−4.0%) and CV (8.1%) in peak velocity, and both the bias (−3.5%) and CV (8.4%) in mean velocity for clinical PC. For rt‐PC, taping an ICD on the abdomen revealed a small bias (−0.3%) and a small CV (3.4%) in peak velocity, and a small bias (−1.8%) in mean velocity. When ICD was taped below the clavicle, both the bias (8.2%) and CV (7.3%) increased in peak velocity, and the bias (3.0%) increased in mean velocity for rt‐PC. Taping an ICD on the abdomen and below the left clavicle led to similar CV (6.4% vs. 5.5%) in mean velocity. Also shown in Figure 3 are the comparisons between rt‐PC with ICD against clinical PC with no ICD. The agreement in peak velocity (CV = 3.8% vs. 4.4%) and the bias in mean velocity (bias = 1.2% vs. 6.2%) progressively worsens when the ICD is placed below the left clavicle. Analysis of variation with Bonferroni correction showed no significant difference (*p* > 0.46) among clinical PC without ICD, clinical PC with ICD, rt‐PC without ICD, and rt‐PC with ICD in peak velocity or mean velocity.

**FIGURE 4:**
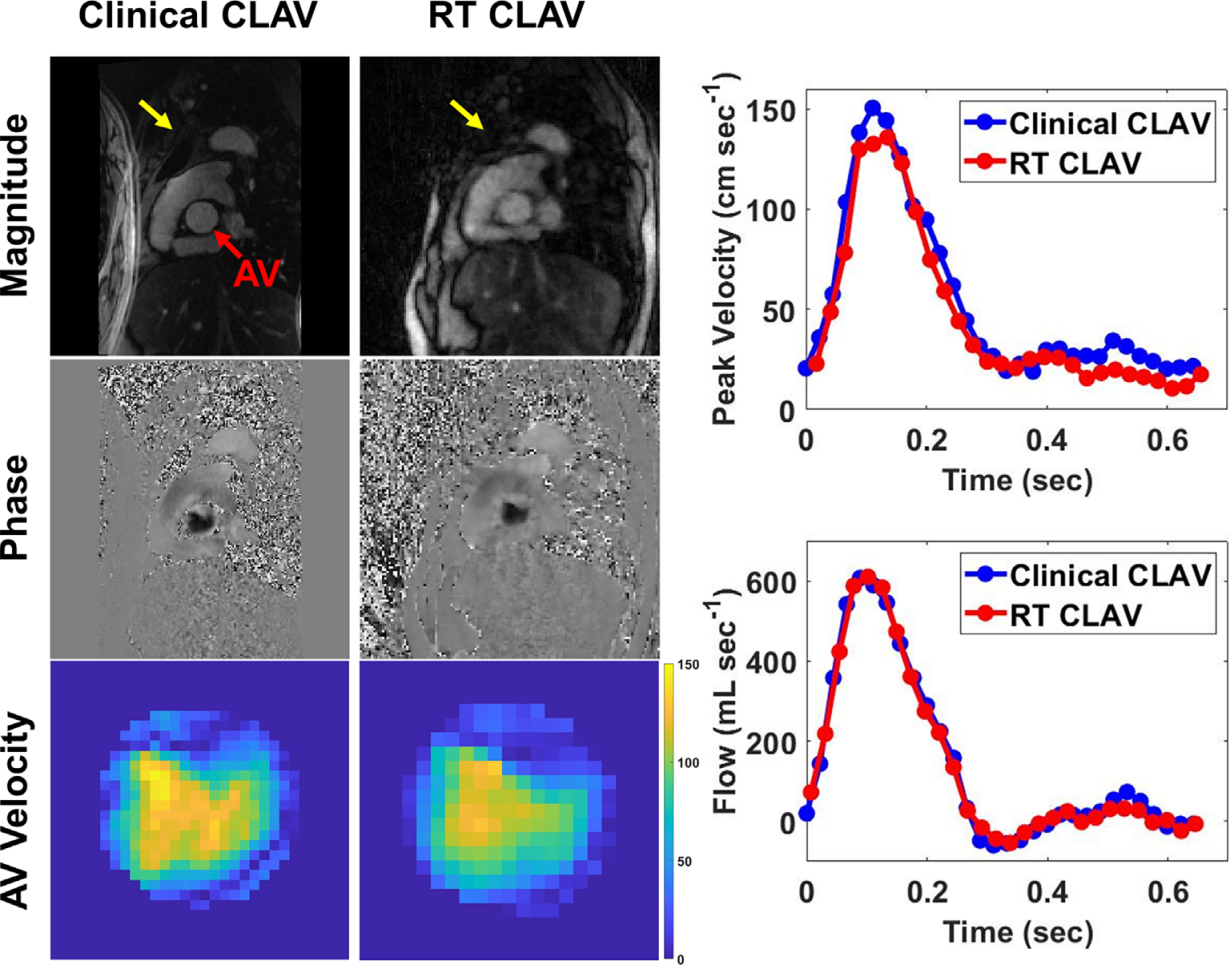
The magnitude images, phase‐difference images, and two‐dimensional aortic velocity profiles at peak systole of a representative adult subject with implantable cardioverter‐defibrillator (ICD) taped below the left clavicle, and the corresponding peak velocity and flow‐time curves above the aortic valve. Phase‐difference images are displayed with grayscale ranging from −π to π. Yellow arrows point to signal voids caused by the ICD. For the corresponding video display, see Video S3. AV, aortic valve; CLAV, with ICD taped below the left clavicle; RT, real time.

Figure 4 shows the magnitude and PC images, 2D aortic velocity profile at peak systole, and the corresponding peak velocity and flow measurements of a representative adult patient with ICD taped below the left clavicle. For dynamic display of images in Figure 4, see Video S3. For the patient shown in Figure 4, the NRMSE (clinical PC vs. rt‐PC) was 6.99% and 2.58% for peak velocity and flow for all time points, respectively, suggesting that both the peak velocity and flow measurements were well matched.

**FIGURE 5:**
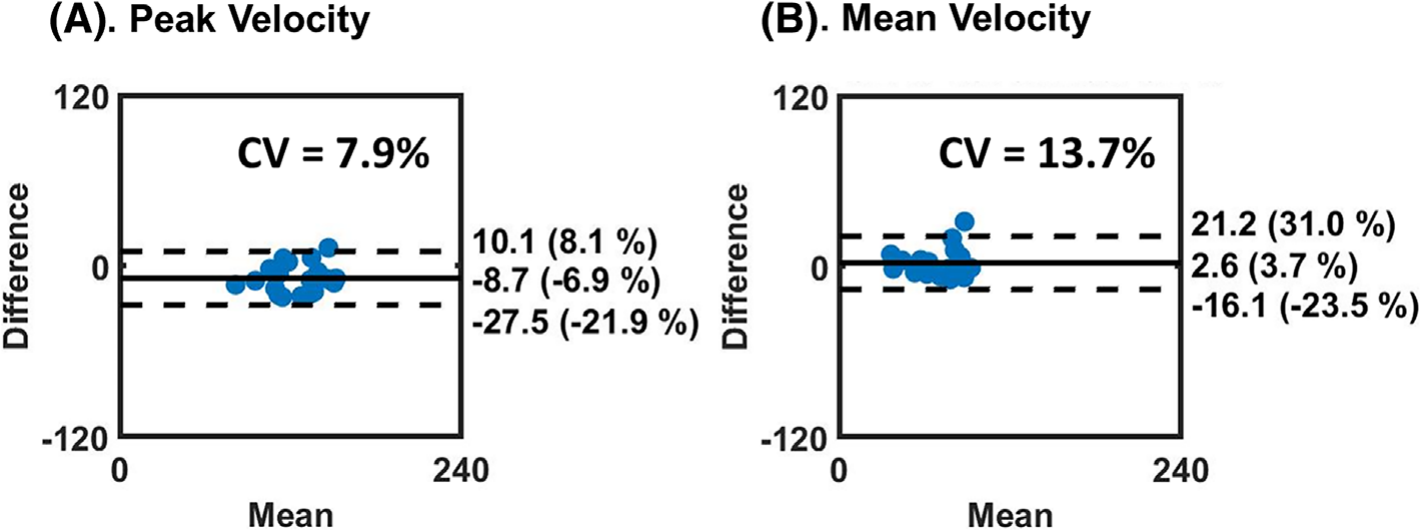
Scatter plots representing the Bland–Altman analysis on peak velocity (A) and mean velocity (B) measurements at peak systole in adults with implantable cardioverter‐defibrillator taped below the left clavicle (real‐time phase contrast vs. clinical phase contrast). Units: cm/s. CV, coefficient of variation.

Figure 5 shows scatter plots illustrating the Bland–Altman analysis on peak and mean velocity measurements at peak systole including all adult subjects with ICD. Real‐time PC achieved good agreement (mean difference = 6.9%; CV = 7.9%) in peak velocity, and good mean difference (3.7%) and moderate CV (13.7%) in mean velocity against clinical PC. In addition to the ICD, ROI contouring also contributed to the CV for mean velocity. A paired two‐tailed t‐test showed no significant difference in mean velocity (*p* > 0.23) and some significant difference in peak velocity (*p* = 0.001) between clinical PC and real‐time PC.

## 4 DISCUSSION

This study evaluates the relative accuracy and precision of clinical standard PC and rt‐PC pulse sequences in both pediatric and adult subjects with an ICD. As demonstrated in the pediatric experiment, both the bias and variation in peak velocity increase as the distance between the ICD and the aortic valve decreases (i.e., abdomen to clavicle). The same trend has been observed in mean velocity for clinical PC. For rt‐PC, the bias increases while variation stays similar as the distance decreases. This trend illustrates that the proximity of the ICD to the heart is an important consideration for ensuring PC‐MRI accuracy. Our results demonstrate that PC MRI is influenced by the presence of an ICD. The extent of its influence is likely determined by a combination of the device size and its proximity to the heart.

This is the first study examining the influence of ICD on the accuracy and precision of PC MRI. For the objective of our study, the ICD taping approach had several advantages over scanning patients with implanted ICDs. First, the taping approach eliminated MR safety concerns and enabled a more comprehensive evaluation beyond the clinical MRI scan times. Second, it enabled comparisons between measurements with and without ICD, where the latter served as the reference. Third, it enabled test–retest experiments. Moreover, despite the taping of the ICD, the results from this study are likely to translate to patients with implanted ICDs. Given that both clinical 2D PC^6^ and four‐dimensional‐flow MRI^10^ have been performed without adverse events in patients with ICDs, rt‐PC is likely to be even safer, given its shorter scan time (˜5 vs. 20 s [standard 2D PC] or 5–10 min [four‐dimensional flow]).

This study has several limitations: (1) We did not test clinical PC or rt‐PC in patients with pacing/high voltage leads. The disruptive effects of leads are likely negligible on the accuracy of velocity and flow measurements through the pulmonic valve, as it is distant from the leads, but their effects on measurements through the tricuspid, aortic, and mitral valves need to be evaluated further. (2) This study did not include subjects with known valvular heart disease. A future study is warranted to evaluate the relative accuracy and precision of rt‐PC in CIED patients with valvular disease, including patients with severe regurgitation. (3) We did not test the effects of different CIED vendors and CIED types (pacemaker, resynchronization devices, subcutaneous ICDs). A future study is warranted to determine whether the findings from this study translate to other CIED vendors and CIED types. (4) We did not investigate the impact of CIED orientation per distance, because that would increase the scan time for each human experiment (i.e., pull patient out of MRI, reposition the device, and rescan). (5) The imaging conditions between rt‐PC and clinical PC were not exact matches. Specifically, rt‐PC data were acquired during free breathing with a flip angle of 15°, whereas clinical PC data were acquired during a breath‐hold with a flip angle of 20°. (6) The signal loss area did not match between rt‐PC (larger due to higher sensitivity to off‐resonance with radial k‐space sampling) and clinical PC. The magnetic field distortion around the ICD may have negatively influenced background phase correction more for rt‐PC than clinical PC. (7) The gadolinium‐doped water used in the flow phantom experiment did not match the viscosity of blood as well as a glycerol mix. (8) The phase‐correction methods for in vivo data were different (first order for clinical PC with Cartesian k‐space sampling vs. second order for rt‐PC with radial k‐space sampling). (9) Finally, because the parent research MRI study included multiple pulse sequences, we did not have time to sample the mitral, tricuspid, or pulmonic valve.

In conclusion, this study demonstrated that aortic velocity measurements derived from 2D‐PC‐MRI pulse sequences are influenced by a CIED in both pediatric and adult subjects with a CIED. The extent of its influence is likely determined by a combination of the device size and its proximity to the heart.

## Supporting information


**Figure S1.** The flow phantom. A U‐shaped polyvinyl chloride (PVC) pipe with inner size 21 mm represents a simplified aorta.
**Figure S2.** Example coronal view of a chest CT of an adult patient with an implantable cardioverter‐defibrillator (ICD). We measured the distance as shown.
**Figure S3.** Anatomic locations where the implantable cardioverter‐defibrillator (ICD) was taped: below the left clavicle in adults (*left column*), below the left clavicle in children (*middle column*), and on the abdomen in children (*right column*).
**Table S1.** Clinical profiles of pediatric (*N* = 8) and adult (*N* = 21) subjects.
**Table S2.** Summary of relevant imaging parameters used in this study.

**Video S1.** Dynamic display of the flow phantom magnitude and phase difference images shown in Figure 1. Left, clinical phase contrast (PC) without implantable cardioverter‐defibrillator (ICD); middle, clinical PC with ICD; right: real‐time PC with ICD. Both clinical PC time series were resampled in spatial and temporal dimensions to match real‐time PC.

**Video S2.** Dynamic display of the magnitude and phase‐difference images from a representative pediatric subject shown in Figure 2. Column 1, Clinical phase contrast (PC) without implantable cardioverter‐defibrillator (ICD); Column 2, clinical PC with ICD taped on the abdomen; Column 3, real‐time PC (rt‐PC) with ICD taped on the abdomen; Column 4, clinical PC with ICD taped below the left clavicle; Column 5, rt‐PC with ICD taped below the left clavicle. The corresponding velocity and flow curves for all time points. Both clinical PC time series were resampled in spatial and temporal dimensions to match the real‐time PC.

**Video S3.** Dynamic display of the magnitude and phase‐difference images from a representative adult subject shown in Figure 4. *Left*: Clinical phase contrast (PC) with implantable cardioverter‐defibrillator (ICD). *Right*: Real‐time PC with ICD. The clinical PC time series was resampled in spatial and temporal dimensions to match the real‐time PC.
