# Supplementary material for: Aortic velocity measurements derived from phase‐contrast MRI are influenced by a cardiac implantable electronic device in both adult and pediatric human subjects
Source: Magn Reson Med. 2024 Dec 6;93(5):2099–107. doi: 10.1002/mrm.30399 (PMC11893034; doi:10.1002/mrm.30399)
Supplement: Supplementary file 1 — Figure S1. The flow phantom. A U‐shaped polyvinyl chloride (PVC) pipe with inner size 21 mm represents a simplified aorta. Figure S2. Example coronal view of a chest CT of an adult patient with an implantable cardioverter‐defibrillator (ICD). We measured the distance as shown. Figure S3. Anatomic locations where the implantable cardioverter‐defibrillator (ICD) was taped: below the left clavicle in adults (left column), below the left clavicle in children (middle column), and on the abdomen in children (right column). Table S1. Clinical profiles of pediatric (N = 8) and adult (N = 21) subjects. Table S2. Summary of relevant imaging parameters used in this study. [file MRM-93-2099-s001.docx]

**Supplementary Materials**

**Appendix**

*Taping a CIED generator to mimic image artifacts*

There are several advantages for mimicking a CIED implantation. First, reference images can be sampled without CIED, which is not possible in patients with implanted CIEDs. Second, it eliminates MR safety concerns associated with scanning patients with implanted CIEDs, recognizing that modern CIEDs have little ferromagnetic materials. In this study, we used an implantable cardioverter-defibrillator because it has a larger generator and causes larger image artifacts (1,2) than a pacemaker (*i.e.*, ICDs produce more artifact than pacemakers). This experimental design does not consider lead artifact from pacing/high voltage leads, but this is not a major detriment since the primary cause of image artifacts is the generator (2).

We measured the mean distance between the center of CIED and heart from the chest CT of 10 de-identified adult patients (mean age = 60.0 ± 11.4 years). While the resulting mean distance was 14 cm, we elected to use 12 cm as the fixed distance between the heart (or phantom) and ICD to be more conservative. Our method to measure the distance from a chest CT is illustrated in Supporting Figure S2.

As shown in Supporting Figure S3, for each pediatric volunteer, an electrophysiologist placed the device based on individualized patient anatomical landmarks (below the left clavicle and on the abdomen). Thus, the resulting distance depended on subject’s body habitus. For each adult patient, the device was taped below the left clavicle (see Supporting Figure S3).

**Supporting Table S1.** Clinical profiles of pediatric (N = 8) and adult (N = 21) subjects.

| **Characteristics (pediatric subjects)** | |
| --- | --- |
| **Age** | **13.5 ± 1.4 years** |
| **Females** | **6 (75%)** |
| **Resting heart rate** | **72.5 ± 7.5 bpm** |
| **LVEF** | **59.0 ± 3.7%** |
| **BMI >30** | **0 (0%)** |
| **Characteristics (adult subjects)** | |
| **Age** | **49.9 ± 14.4 years** |
| **Females** | **9 (42.9%)** |
| **Resting heart rate** | **65.3 ± 10.2 bpm** |
| **LVEF** | **58.5 ± 6.6%** |
| **Diabetes** | **4 (19.1%)** |
| **Hypertension** | **9 (42.9%)** |
| **BMI >30** | **11 (52.4%)** |
| **History of smoking** | **5 (23.8%)** |
| **History of atrial fibrillation** | **1 (4.8%)** |

**Supporting** **Table S2.** Summary of relevant imaging parameters used in this study. Clinical PC acquisitions for both the adult and pediatric experiments involving an ECG signal were conducted with retrospective ECG-gating with inline temporal interpolation to reconstruct 30 cardiac frames, whereas clinical PC acquisition for the phantom experiment involving an external trigger pulse was conducted with prospective ECG-triggering without temporal interpolation. This discrepancy was due to the exclusion of retrospective ECG-gating option in the clinical PC sequence when using an external trigger pulse emitted by the LVAD pump system.

| **Fixed parameters for adults, children, and phantom** | | |
| --- | --- | --- |
|  | **Clinical PC** | **Real-time PC** |
| **Acceleration factor** | **2** | **64** |
| **Slice Thickness (mm)** | **6** | **7** |
| **Flip Angle (^o^)** | **20** | **15** |
| **Receiver Bandwidth (Hz/Pixel)** | **450** | **793** |
| **Specific parameters for children** | | |
| **Field of View (mm^2^)** | **300 x 206 to 340 x 234** | **340 x 340** |
| **Acquisition Matrix** | **192 x 132** | **160 x 160** |
| **Spatial Resolution (mm^2^)** | **1.6 x 1.6 to 1.8 x 1.8** | **2.1 x 2.1** |
| **TE (ms)** | **2.47 to 2.5** | **2.58** |
| **TR (ms)** | **4.44 to 4.54** | **3.94** |
| **Temporal Resolution (ms)** | **37.1 to 37.8** | **23.64** |
| **Velocity Encoding (cm/sec)** | **150** | **150** |
| **Number of phases per heartbeat** | **30 (temporal interpolation)** | **211** |
| **k-space lines per frame per heartbeat** | **4 (segmented)** | **3 (single-shot)** |
| **Scan duration (heartbeats)** | **17** | **5** |
| **Specific parameters for adults** | | |
| **Field of View (mm^2^)** | **340 x 231 to 379 x 262** | **400 x 400** |
| **Acquisition Matrix** | **192 x 132** | **160 x 160** |
| **Spatial Resolution (mm^2^)** | **1.78 x 1.78 to 1.98 x 1.98** | **2.5 x 2.5** |
| **TE (ms)** | **2.47** | **2.58** |
| **TR (ms)** | **4.6** | **3.94** |
| **Temporal Resolution (ms)** | **37.12** | **23.64** |
| **Velocity Encoding (cm/sec)** | **150** | **150** |
| **Number of phases per heartbeat** | **30 (temporal interpolation)** | **211** |
| **k-space lines per frame per heartbeat** | **4 (segmented)** | **3 (single-shot)** |
| **Scan duration (heartbeats)** | **17** | **5** |
| **Specific parameters for the flow phantom** | | |
| **Field of View (mm^2^)** | **300 x 206** | **300 x 300** |
| **Acquisition Matrix** | **192 x 132** | **160 x 160** |
| **Spatial Resolution (mm^2^)** | **1.6 x 1.6** | **1.9 x 1.9** |
| **TE (ms)** | **2.91** | **2.25** |
| **TR (ms)** | **5.08** | **4.61** |
| **Temporal Resolution (ms)** | **30.48** | **27.70** |
| **Velocity Encoding (cm/sec)** | **60** | **60** |
| **Number of phases per heartbeat** | **32** | **180** |
| **k-space lines per frame per heartbeat** | **3 (segmented)** | **3 (single-shot)** |
| **Scan duration (heartbeats)** | **22** | **5** |


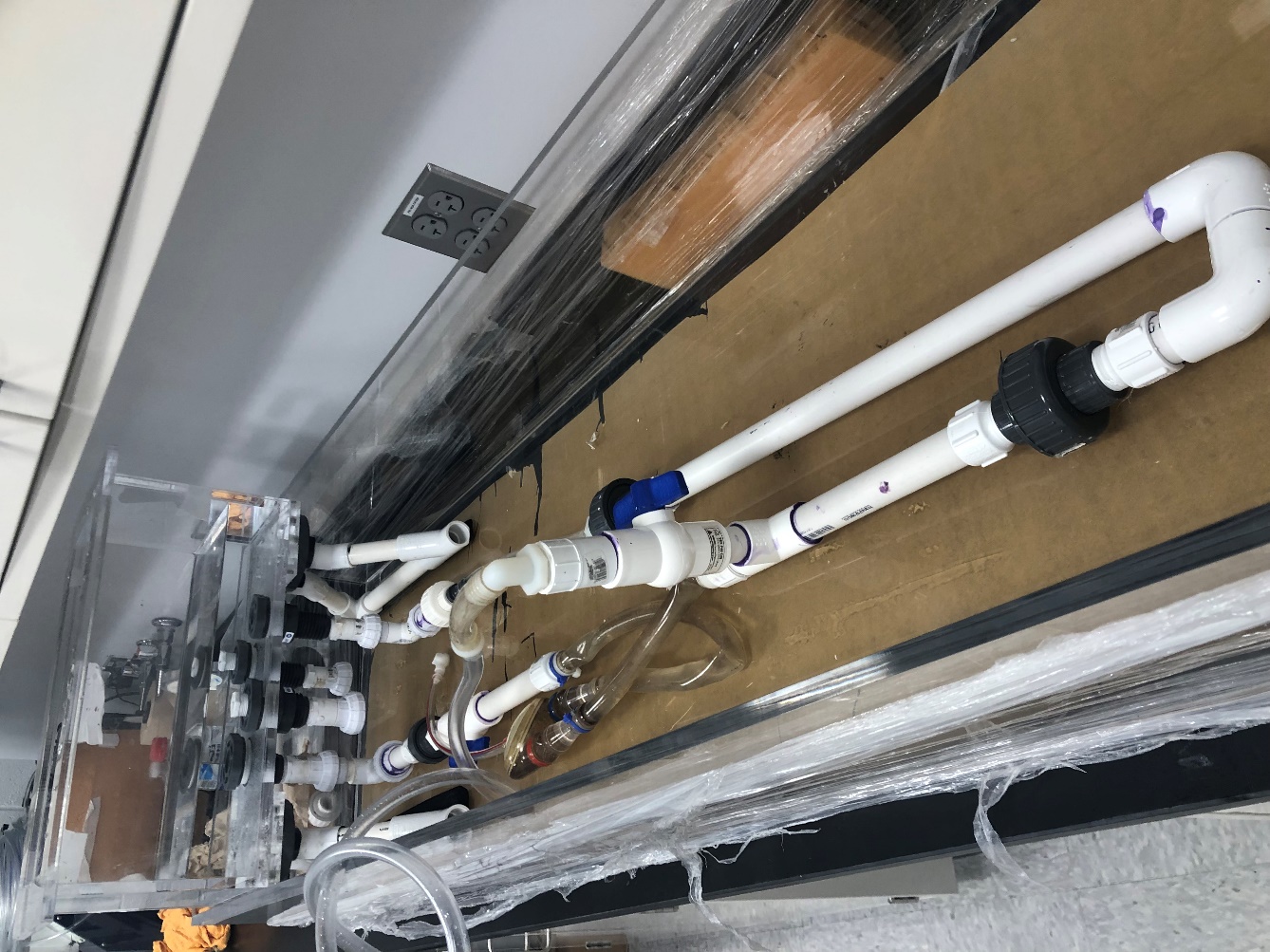
**Supporting Figure S1.** The flow phantom. A U-shaped PVC pipe with inner size 21 mm represents a simplified aorta.


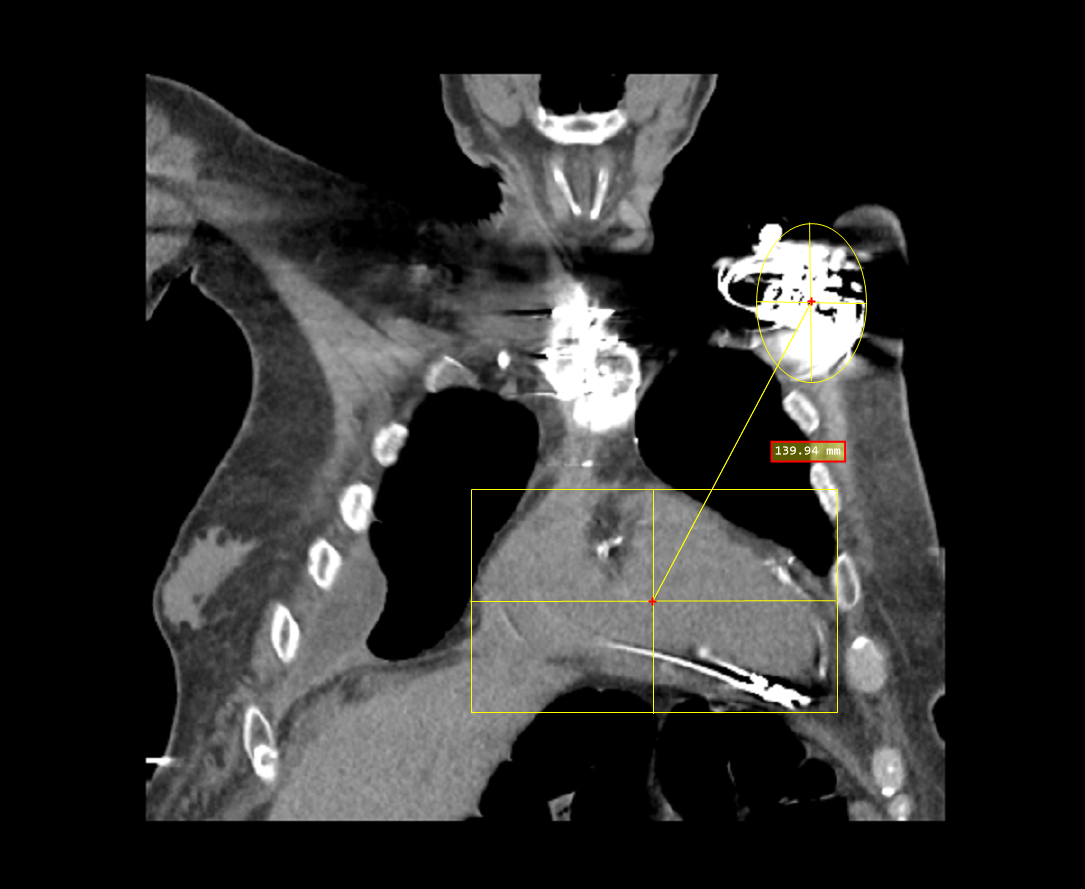


**Supporting Figure S2.** Example coronal view of a chest CT of an adult patient with a CIED. We measured the distance as shown.


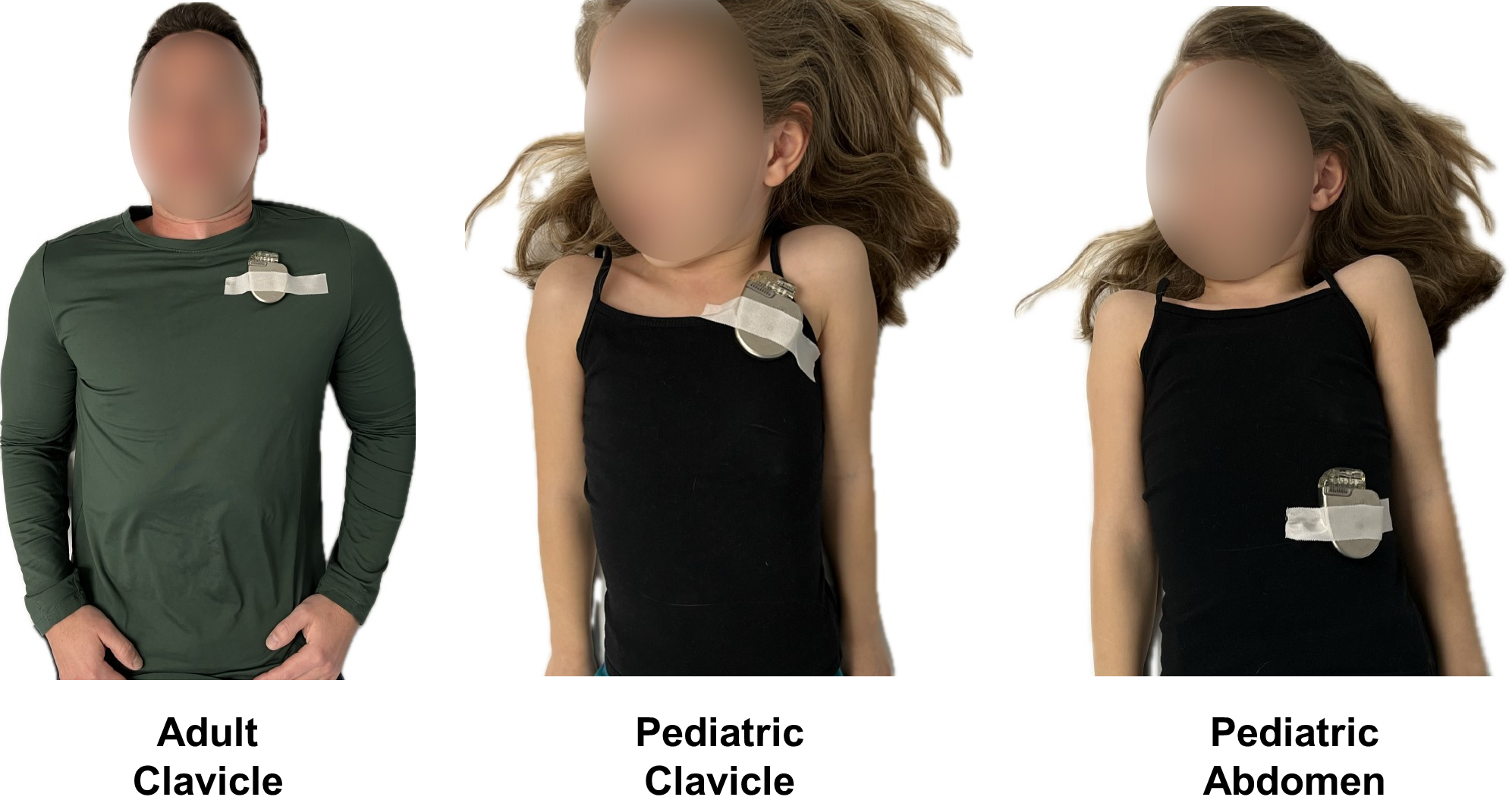


**Supporting Figure S3.** Anatomic locations where IPG was taped: below the left clavicle in adults (left column), below the left clavicle in children (middle column), and on the abdomen in children (right column).

**Supporting Video S1.** Dynamic display of the flow phantom magnitude and phase difference images shown in Figure 1. Left, clinical PC without IPG; middle, clinical PC with IPG; right: real-time PC with IPG. Both clinical PC time series were resampled in spatial and temporal dimensions to match real-time PC.

**Supporting Video S2.** Dynamic display of the magnitude and phase difference images from a representative pediatric subject shown in Figure 2. Column 1: clinical PC without IPG; column 2: clinical PC with IPG taped on the abdomen; column 3: rt-PC with IPG taped on the abdomen; column 4: clinical PC with IPG taped below the left clavicle; column 5: rt-PC with IPG taped below the left clavicle. The corresponding velocity and flow curves for all time points. Both clinical PC time series were resampled in spatial and temporal dimensions to match real-time PC.

**Supporting Video S3.** Dynamic display of the magnitude and phase difference images from a representative adult subject shown in Figure 4. Left, clinical PC with IPG; right: real-time PC with IPG. Clinical PC time series was resampled in spatial and temporal dimensions to match real-time PC.

**References**

1. Hilbert S, Jahnke C, Loebe S, Oebel S, Weber A, Spampinato R, Richter S, Doering M, Bollmann A, Sommer P, Hindricks G, Paetsch I. Cardiovascular magnetic resonance imaging in patients with cardiac implantable electronic devices: a device-dependent imaging strategy for improved image quality. Eur Heart J Cardiovasc Imaging 2018;19(9):1051-1061.

2. Sasaki T, Hansford R, Zviman MM, Kolandaivelu A, Bluemke DA, Berger RD, Calkins H, Halperin HR, Nazarian S. Quantitative assessment of artifacts on cardiac magnetic resonance imaging of patients with pacemakers and implantable cardioverter-defibrillators. Circulation Cardiovascular imaging 2011;4(6):662-670.
